# Therapeutic Potential of Mitochondrial Transplantation with Focus on DBD

**DOI:** 10.3390/ijms27083379

**Published:** 2026-04-09

**Authors:** Chen Guo, Chenwei Gu, Anjie Chen, Sixin Li, Si Shen, Zhonghao Tang, Jiandong Gui, Lijie Zhu, Sheng Wu, Yuanyuan Mi

**Affiliations:** 1 Department of Urology, Affiliated Hospital of Jiangnan University, 1000 Hefeng Road, Wuxi 214122, China; gc44223682@gmail.com (C.G.); njmugcw@163.com (C.G.); alexchenmed964@gmail.com (A.C.); leesixin123@163.com (S.L.); 13921101627@163.com (S.S.); tangzhonghao0108@163.com (Z.T.); gui1996xx@163.com (J.G.); jndxfyzlj@163.com (L.Z.); wusheng7012@sina.com (S.W.); 2 Wuxi School of Medicine, Jiangnan University, 1800 Lihudadao, Wuxi 214122, China

**Keywords:** diabetic bladder disease, mitochondrial transplantation, mitochondrial dysfunction, oxidative stress

## Abstract

Diabetic Bladder Disease (DBD), a common urological complication of diabetes mellitus, severely compromises the quality of life of affected patients. Mitochondria, the primary energy-producing organelles in cells, are closely correlated with the pathogenesis and progression of DBD. As an emerging therapeutic modality, mitochondrial transplantation exhibits substantial potential for the management of DBD. This paper presents a comprehensive review of mitochondrial transplantation, with a focus on its fundamental theories, application conditions, safety profiles, and mitochondrial sources. Subsequently, we explore the association between mitochondrial dysfunction and the pathological mechanisms underlying DBD, analyze the disparities between mitochondrial transplantation and conventional therapeutic approaches, and discuss the prospects of combined and personalized treatment regimens. Finally, this review summarizes the ethical controversies surrounding this therapeutic strategy and outlines future research trends, aiming to lay a theoretical foundation for the development of novel therapeutic modalities against DBD.

## 1. Introduction

DBD, a prevalent chronic complication of diabetes mellitus, arises primarily from persistent hyperglycemia-mediated damage. It exhibits a high incidence among diabetic patients, with estimates indicating that approximately 50–80% of individuals with diabetes experience varying degrees of bladder dysfunction [1]. Hyperglycemia directly impairs the pelvic autonomic nerves, triggering neuropathy [2]; it also disrupts the metabolism and contractile function of bladder smooth muscle cells (BSMCs), induces bladder wall fibrosis [3], and, in conjunction with pelvic angiopathy-induced local hypoperfusion, collectively impairs the sensory transmission and motor regulation mechanisms of the bladder [4]. Pathologically, DBD is characterized by progressive deterioration of bladder function. In the early stage, it mainly manifests as bladder hypoesthesia, reduced micturition frequency, and increased residual urine volume [2]. However, emerging clinical -evidence in recent years has demonstrated that it can also present with storage symptoms such as urinary urgency and frequency [5]. As the disease advances, patients may gradually develop dysuria and a sensation of incomplete bladder emptying, which can progress to urinary retention and recurrent urinary tract infections in severe cases [6,7]. Over the long term, complications including excessive bladder distension and vesicoureteral reflux can lead to renal impairment and even renal failure [8]. Notably, a subset of patients predominantly exhibits detrusor overactivity symptoms such as frequent micturition, urgency, and nocturia, which significantly disrupt the homeostasis of the urinary system and compromise patients’ quality of life.

Mitochondria are core organelles with a double-membrane structure in eukaryotic cells. As the primary source of cellular energy, mitochondria generate approximately 95% of the energy required for cellular life activities through the oxidative phosphorylation (OXPHOS) process of aerobic respiration [9]. Meanwhile, they are deeply involved in the metabolic regulation of fats, amino acids, and carbohydrates [10]. Moreover, mitochondria participate in cellular signal transduction and apoptotic processes by regulating calcium ion homeostasis and releasing apoptosis-inducing factors, among other mechanisms [11,12]. Thus, they are pivotal organelles for maintaining cellular physiological functions and homeostasis.

The pathogenesis and progression of DBD are closely associated with mitochondrial dysfunction. As the energy powerhouse of bladder tissue cells, impaired mitochondrial function represents one of the key mechanisms underlying this diabetic complication [13]. Chronic diabetic conditions such as sustained hyperglycemia and insulin resistance induce mitochondrial damage in bladder detrusor muscle cells, urothelial cells, and peripheral nerve cells [14]. This damage is characterized not only by ultrastructural alterations including mitochondrial swelling, decreased matrix density, and fractured or sparse cristae, but also by severe energy metabolism disorders. Specifically, the suppression of OXPHOS and obstruction of the tricarboxylic acid cycle lead to insufficient ATP production, which impairs the contractile function of the bladder detrusor muscle and consequently manifests as clinical symptoms such as dysuria and increased residual urine volume [15]. Meanwhile, damaged mitochondria overproduce reactive oxygen species (ROS), triggering oxidative stress responses and activating apoptotic pathways and inflammatory signaling cascades [16]. These processes exacerbate bladder tissue injury and fibrosis, driving disease progression from the compensated stage to the decompensated stage. Therefore, we propose that improving mitochondrial function constitutes a critical therapeutic strategy for the intervention of DBD.

## 2. Mitochondrial Transplantation

### 2.1. The Biological Basis of Mitochondrial Transplantation

Mitochondrial transplantation is an advanced biomedical technology that involves introducing healthy and functionally competent mitochondria from donors (e.g., stem cells and mature somatic cells) into recipient cells, tissues or living organisms to replace or supplement impaired mitochondria and rectify energy metabolism deficits [17]. Its essence lies in “organelle-level repair and replacement”, which entails replenishing functional mitochondria without altering the nuclear genome of recipient cells [18]. Mitochondrial transplantation is underpinned by solid biological rationale. As the core hub of cellular energy metabolism, mitochondria are also implicated in a variety of essential cellular processes, including metabolic regulation, homeostasis maintenance, signal transduction and organelle quality control [19]. Notably, intercellular mitochondrial transfer has been identified in experimental studies, which provide critical theoretical support for the development of mitochondrial transplantation. For instance, co-culture experiments have verified that mesenchymal stem cells (MSCs) are capable of transferring mitochondria to damaged cells, thereby ameliorating their biological functions [20]. To date, a diverse array of mitochondrial transplantation techniques has been developed. Direct mitochondrial transplantation involves isolating and purifying mitochondria from healthy cells, then directly introducing them into damaged cells via physical or chemical methods such as microinjection and pressure-driven delivery, thereby achieving direct supplementation of exogenous functional mitochondria [21,22]. Cell-mediated mitochondrial transfer uses living cells such as stem cells as carriers and realizes active mitochondrial transport from donor cells to damaged cells through tunnel nanotubes and other structures via co-culture or in vivo transplantation [23]. In addition, extracellular vesicle-mediated mitochondrial transfer employs vesicles including exosomes as delivery vehicles to transfer mitochondrial components to target cells for the repair of mitochondrial function [24]. A representative example is MitoPunch, a pressure-driven mitochondrial transfer device that enables the simultaneous delivery of isolated mitochondria into a large number of target mammalian cells, yielding cell populations stably harboring exogenous mitochondrial DNA (mtDNA) [25]. Another cutting-edge approach is based on FluidFM technology, which allows the extraction, injection and transplantation of organelles from living cells with subcellular spatial resolution. This technique facilitates precise intercellular mitochondrial transplantation, and the transplanted mitochondria can integrate into the mitochondrial network of host cells [26]. The advent of these technologies has furnished practical technical tools for the clinical translation and experimental implementation of mitochondrial transplantation.

### 2.2. Analysis of the Conditions and Safety of Mitochondrial Transplantation

The conditions for mitochondrial transplantation constitute the foundation for its technical application. The primary consideration is the compatibility between transplanted mitochondria and the host. To mitigate the risk of immune recognition, priority should be given to selecting donors with high homology, such as those from conspecifics or inbred strains. Simultaneously, it is imperative to ensure that donors provide an adequate quantity of healthy mitochondria, which are free from bacterial and viral contamination, with donor cell debris and cytoplasmic proteins removed to minimize foreign body stimulation. For recipients, they must be free from severe immune hyperactivity or immunodeficiency to avoid compromising the survival of transplanted mitochondria; additionally, they should not have severe organ failure, thus ensuring that target tissues can respond normally to energy metabolism restoration [27]. On this basis, the targeting ability and delivery efficiency of mitochondrial transplantation need to be guaranteed to enhance the credibility and feasibility of related experiments [28].

Safety is a pivotal issue for the clinical translation of mitochondrial transplantation. Studies have demonstrated that neither direct nor indirect, acute nor chronic allogeneic reactions, allorecognition, or damage-associated molecular pattern (DAMP) responses are detectable in animal experiments, regardless of whether autologous or allogeneic mitochondrial transplantation is performed [29]. In porcine models of myocardial ischemia–reperfusion (IR) injury, intracoronary delivery of mitochondria has been proven safe; it also significantly increases coronary blood flow, improves myocardial function and perfusion, and reduces infarct size [30]. In porcine models of acute kidney injury (AKI), intra-arterial injection of autologous mitochondria has not been associated with any safety concerns while also enhancing renal function and alleviating kidney damage [31]. Nevertheless, mitochondrial transplantation may be confronted with certain potential risks. For instance, exogenous mitochondria are susceptible to damage in the extracellular environment, which may subsequently trigger adverse danger signals. Studies have found that isolated mitochondria are prone to modification by advanced glycation end products (AGEs), whereas O-GlcNAcylation modification can prevent mitochondrial glycosylation and improve the therapeutic efficacy of transplanted mitochondria in the central nervous system [32]. Furthermore, donor mitochondrial quality control is challenging to implement. If donor cells harbor occult pathological changes, genetic defects, contamination, or other abnormalities, these issues may be transmitted to recipients via transplantation, thereby inducing novel cellular dysfunction [27]. Overall, although current research indicates that mitochondrial transplantation exhibits a certain level of safety, long-term monitoring and more clinical studies are still required to comprehensively evaluate its safety profile and potential side effects.

### 2.3. Sources of Mitochondrial Isolation and Clinical Trial Design

Donor cell sources for mitochondrial transplantation are extensive, yet all must strictly adhere to the principle of “high yield, high activity, and low risk” [33]. Currently, MSCs—including those derived from bone marrow, umbilical cord, and adipose tissue—are the primary preferred option in clinical research. Owing to their advantages such as abundant mitochondrial yield, low immunogenicity, convenient in vitro expansion, and robust functional stability, MSCs are widely applicable for treating diverse disorders including myocardial ischemia and neurodegenerative diseases [34]. In addition, skeletal muscle cells represent an important choice for autologous transplantation and emergency settings, attributed to their high mitochondrial density, non-invasive autologous acquisition, and complete homology matching [35]. Induced pluripotent stem cells (iPSCs) and their directionally differentiated cells (e.g., cardiomyocytes, neurons) have emerged as a cutting-edge avenue for personalized therapy, characterized by excellent immunocompatibility and strong functional adaptability [36]. Furthermore, functionally specialized cells such as hepatocytes and macrophages can supply mitochondria tailored to targeted diseases [37].

Following the selection of suitable mitochondria, an equally crucial step is to obtain and ensure sufficiently purified mitochondrial isolates. At present, the standard protocol for basic purification is differential centrifugation, which separates substances with varying densities by applying different centrifugal speeds to isolate mitochondria [38]. Density gradient centrifugation involves using media such as Percoll or sucrose to form a continuous density gradient, where mitochondria form distinct bands based on density differences, enabling precise isolation [39]. In addition, there is filtration purification, a method that physically sieves out impurities larger than mitochondria using filters with different pore sizes. However, its standalone application yields low purity, thus necessitating combination with other optimization methods [40]. Another approach is immunoaffinity purification, which achieves targeted isolation and purification by leveraging the binding between specific surface antigens of mitochondria (e.g., translocase of outer mitochondrial membrane 22 (Tom22) and cytochrome c oxidase subunit IV (COX IV)) and magnetic beads conjugated with corresponding antibodies [41].

**Figure 1:**
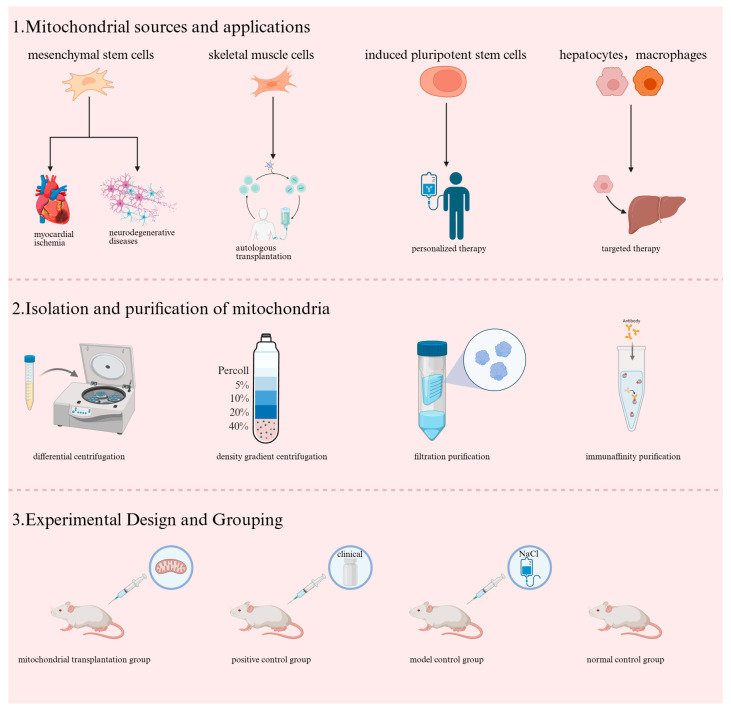
This figure elaborates on the current types of mitochondrial sources, the methods for their acquisition and purification, as well as the grouping strategies for mitochondrial transplantation experimental designs.

After obtaining purified mitochondria, the design of compatible clinical trials requires comprehensive consideration of multiple factors. Taking oocyte mitochondrial transplantation-related research as an example, when investigating mitochondrial function and transplantation efficacy in aged mouse oocytes in vivo, superovulated oocytes collected at different time points were divided into fresh and aged groups. Functional parameters including mitochondrial membrane potential and oxygen consumption rate were evaluated; subsequently, somatic cell mitochondria were microinjected into aged oocytes to observe fertilization rate and embryonic development potential [42]. In MSC-related studies, MSCs labeled with MitoTracker were co-cultured with U87-MG cells or rat cardiomyocytes. Flow cytometry analysis and fluorescence imaging were employed to assess the mitochondrial transfer capacity of MSCs, along with measurements of mitochondrial ROS levels and mitochondrial respiratory parameters [43]. For diabetes-associated research, similar design principles can be adopted. Appropriate DBD patients or animal models should be selected, with groups set as follows: mitochondrial transplantation group (mitochondria administered post-modeling), positive control group (clinically used drugs administered post-modeling), model control group (normal saline administered post-modeling), and normal control group (no modeling performed). The effects of mitochondrial transplantation on bladder function-related indicators (e.g., urodynamic parameters) should be observed, while simultaneously monitoring the in vivo distribution, metabolism, and interactions of transplanted mitochondria with host cells, so as to comprehensively evaluate the therapeutic efficacy of mitochondrial transplantation (Figure 1).

### 2.4. Recent Advances in Clinical Studies of Mitochondrial Transplantation

In recent years, mitochondrial transplantation has rapidly transitioned from basic research to clinical translation owing to its unique advantages of directly supplementing healthy mitochondria and restoring cellular energy metabolism, emerging as an innovative therapeutic strategy for multi-system refractory diseases. A number of early-stage clinical trials have been conducted worldwide to evaluate its safety and efficacy across diverse disease areas. Boston Children’s Hospital initiated a clinical trial intending to recruit 16 pediatric cardiac patients under 18 years of age who were supported by ECMO and presented with myocardial ischemia–reperfusion injury. Healthy mitochondria were extracted from the patients’ autologous chest wall skeletal muscle and transplanted into ischemic myocardium via either surgical injection or cardiac catheter-based infusion. The trial had a primary short-term safety objective and secondary short-term efficacy objectives: the former aimed to observe the incidence of severe adverse events within one week after transplantation to verify the acute clinical safety of the technique; the latter evaluated the reparative effect of mitochondrial transplantation on myocardial function and its role in facilitating weaning by measuring changes in left ventricular ejection fraction from 1 week to 1 month after transplantation and recording the time to successful liberation from ECMO [44]. Paean Biotechnology launched a trial for patients with refractory polymyositis or dermatomyositis. Nine patients aged 19 years or older who were refractory or intolerant to conventional therapy received a single intravenous infusion of mitochondrial product PN-101 derived from allogeneic umbilical cord mesenchymal stem cells. The primary objective was to determine the maximum tolerated dose (MTD) of PN-101 and assess safety by monitoring the incidence of dose-limiting toxicities (DLT) within two weeks after administration. Secondary objectives were to explore efficacy using the IMACS-TIS score for muscular improvement, CDASI and PPNRS for cutaneous lesions and pruritus, and CSAM for core disease activity measures, aiming to provide a safe dose and preliminary efficacy evidence of mitochondrial transplantation for refractory myositis [45]. In addition, Minovia Therapeutics conducted a trial enrolling seven pediatric patients aged 3–18 years with Pearson syndrome caused by mtDNA defects. The patients received mitochondrial augmentation therapy consisting of a single intravenous infusion of autologous CD34+ hematopoietic stem cells ex vivo enriched with healthy donor-derived mitochondria. Over a 12-month follow-up period, the study primarily evaluated treatment-related adverse events to confirm safety and assessed global functional improvement using the IPMDS score. It aimed to validate the safety and clinical efficacy of this innovative mitochondrial transplantation regimen for the rare mitochondrial disorder Pearson syndrome and provide early clinical evidence for cell-based combined mitochondrial augmentation therapy in mitochondrial diseases [46]. IVI Valencia in Spain enrolled 59 women aged 18–42 years with previous in vitro fertilization (IVF) failure due to poor embryo quality. Using an adaptive design, retrieved metaphase II oocytes from each patient were randomly assigned to two groups: the experimental group received microinjection of autologous mitochondria from ovarian stem cells during intracytoplasmic sperm injection (ICSI), while the control group underwent standard ICSI alone. Embryo quality was assessed by morphological, morphokinetic, and preimplantation genetic screening criteria, with the ongoing pregnancy rate at 12 weeks as the primary endpoint. The study aimed to verify whether autologous mitochondrial transplantation as an adjunct to ICSI could improve oocyte and embryo quality and enhance pregnancy outcomes in patients with refractory infertility [47]. Although mitochondrial transplantation remains in the early exploratory stage and key issues including delivery routes, dosage standards, and long-term efficacy require further optimization, these studies firmly establish it as a novel cross-disciplinary, multi-disease therapeutic modality. It holds substantial promise as a new solution for refractory diseases that respond poorly to conventional treatments.

## 3. Feasibility of Mitochondrial Transplantation for the Treatment of DBD

### 3.1. Pathological Metabolic Abnormalities and Pathogenesis of DBD

**Figure 2:**
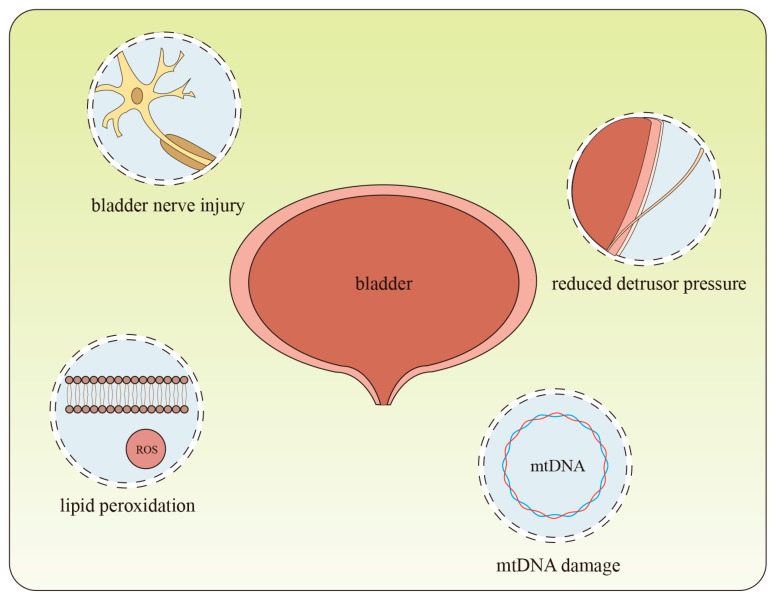
This image illustrates the pathogenesis of bladder disease, which can be induced through mechanisms including lipid peroxidation, reduced detrusor pressure, mtDNA damage, and bladder nerve injury.

In DBD, oxidative stress is recognized as a crucial factor triggering hyperglycemia-dependent tissue damage, and it also plays a pivotal role in the pathological progression of DBD [14,48]. Studies have demonstrated that elevated oxidative stress levels in diabetic patients lead to excessive production of ROS in bladder tissues, which induces lipid peroxidation as well as protein and nucleic acid damage, thereby impairing the normal physiological functions of the bladder. Research has shown that administration of grape seed proanthocyanidin extract (GSPE) can ameliorate bladder function in diabetic rats, a mechanism associated with the regulation of antioxidant enzyme activities (e.g., superoxide dismutase (SOD) and glutathione peroxidase (GSH-Px)) and the alleviation of oxidative stress [49]. Meanwhile, oxidative stress related to metabolic syndrome can induce bladder detrusor fibrosis and detrusor underactivity. In the Ossabaw pig model, the expression levels of oxidative stress- and fibrosis-related biomarkers in bladder tissues were significantly increased in the metabolic syndrome group, accompanied by a reduction in detrusor pressure [50]. Increased cellular apoptosis represents another hallmark of abnormal cellular metabolism in DBD. In diabetic mouse models, smooth muscle-specific knockout of manganese superoxide dismutase (MnSOD) exacerbated diabetes-induced bladder dysfunction, which was accompanied by upregulated expression of apoptosis-related proteins Bax and cleaved caspase-3, as well as downregulated expression of Bcl-2. These findings indicate that oxidative stress can accelerate the progression of bladder dysfunction by activating apoptotic pathways [51]. Diabetes-induced oxidative stress can activate poly(ADP-ribose) polymerase (PARP), which in turn triggers the c-Jun N-terminal kinase (JNK) signaling pathway and the mitochondrial apoptotic pathway, leading to increased apoptosis of bladder cells. This mechanism has been verified in streptozotocin-induced diabetic rat models [52]. Additionally, diabetes can cause mtDNA damage and reduced metabolic flexibility, impairing normal mitochondrial functions. For instance, abnormal mtDNA content and transcription have been observed in both patients with diabetic nephropathy and glomerular mesangial cells cultured in high-glucose conditions [53]. Given that DBD and diabetic nephropathy are both classified as diabetic microangiopathies, similar outcomes regulated by hyperglycemia-induced mitochondrial damage may also be identified in DBD [53]. In diabetic mouse models, various diabetes-related DAMPs can also activate the NLRP3 inflammasome in bladder urothelial cells, triggering inflammatory responses. This process further results in alterations in bladder nerve density and the number of specific nerve fibers (Aδ fibers and C fibers), ultimately causing bladder nerve injury and the onset of DBD [54] (Figure 2).

### 3.2. Mechanisms Underlying the Therapeutic Effects of Mitochondrial Transplantation in Diabetic Bladder Dysfunction

Mitochondrial transplantation exerts therapeutic effects on DBD through four interconnected mechanisms: attenuating oxidative stress, restoring mitochondrial energy metabolism, repairing neural function, and maintaining calcium homeostasis. Together, these pathways synergistically promote the recovery of bladder structure and physiological function.

**Figure 3:**
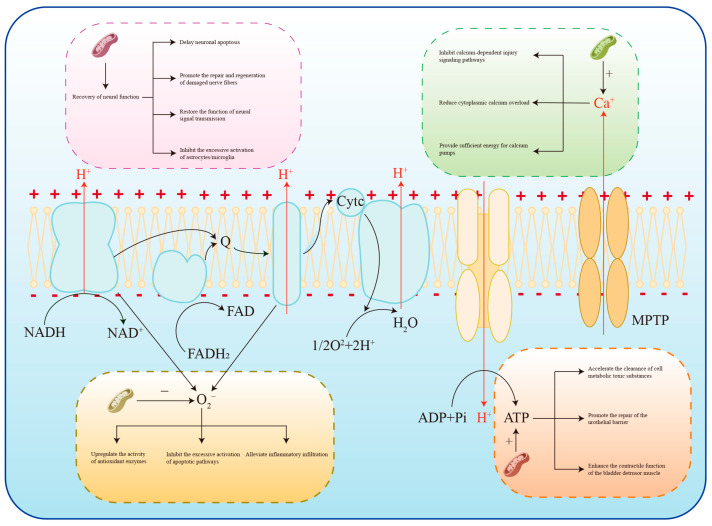
This figure elaborates on the therapeutic pathways of mitochondrial transplantation for DBD. Specifically, it regulates ROS by upregulating the activity of antioxidant enzymes, inhibiting the excessive activation of apoptotic pathways, and alleviating inflammatory infiltration; restores ATP production by accelerating the clearance of cellular metabolic toxic substances, promoting the repair of the urothelial barrier, and enhancing the contractile function of the bladder detrusor muscle; reinstates calcium homeostasis through inhibiting calcium-dependent injury signaling pathways, reducing cytoplasmic calcium overload, and providing sufficient energy for calcium pumps; and facilitates the recovery of DBD by restoring bladder neural function—including promoting neural function recovery, delaying neuronal apoptosis, enhancing the repair and regeneration of damaged nerve fibers, restoring neural signal transmission function, and inhibiting the excessive activation of astrocytes/microglia. (The “+” and “−” symbols on the lines indicate promotion and inhibition of the corresponding factors, respectively. The arrows represent the reaction direction of respiratory chain participants and illustrate the pathways through which they participate in repairing DBD during mitochondrial transplantation).

Mitochondria are the primary source of ROS. Under pathological conditions, dysfunctional mitochondria generate excessive ROS, reduce nitric oxide (NO) bioavailability, trigger inflammatory responses and vasoconstriction, damage cellular macromolecules, and accelerate apoptosis. Mitochondrial transplantation can directly intervene in oxidative stress by replenishing intact functional mitochondria. Pang et al. [55] reported that, in a mouse model of acute kidney injury induced by hemorrhagic shock combined with rhabdomyolysis, intravenously injected allogeneic healthy mitochondria accumulated in injured renal tissue; upregulated the expression of SOD1, SOD2, Nrf2, and HO-1; decreased the BAX/Bcl-2 ratio and cleaved caspase-3 levels; inhibited tubular epithelial cell apoptosis; and preserved renal function. In the pathogenesis of DBD, the hyperglycemic microenvironment induces mitochondrial dysfunction and excessive ROS production, thereby triggering inflammation, apoptosis, and extracellular matrix deposition. IR-61, a mitochondria-targeted antioxidant, activates the Nrf2/Keap1 pathway in bladder smooth muscle cells; upregulates HO-1, SOD1/2, and GPX1; alleviates oxidative stress and mitochondrial damage; suppresses apoptosis; and maintains detrusor contractility [15]. These mechanisms are highly consistent with the biological effects of mitochondrial transplantation, supporting its translational potential for DBD treatment (Figure 3).

As the main producer of ATP via OXPHOS, injured mitochondria severely disrupt cellular energy supply. Kim et al. [56] demonstrated that in idiopathic inflammatory myopathy, exogenous mitochondria (PN-101) restored the expression of mitochondrial respiratory chain proteins and improved cell viability. Intravenous administration of PN-101 also enhanced muscle strength in mouse models and clinical patients. Iwata et al. [57] found that exogenous mitochondria increased ATP production in the hippocampus and improved depression- and anxiety-like behaviors. In schizophrenia models, mitochondrial transplantation elevated neuronal mitochondrial membrane potential and restored dendritic arborization and synaptic structure. In DBD, mitochondrial dysfunction leads to insufficient ATP synthesis, resulting in detrusor underactivity, decreased contractility, and progressive fibrosis. Mitochondrial transplantation replenishes intact mitochondria, restores OXPHOS efficiency and ATP generation, supports detrusor contraction and urothelial barrier repair, accelerates metabolic waste clearance, inhibits fibrogenesis, and improves smooth muscle contractile coordination. Li et al. [16] reported that oridonin activates AMPK/PINK1/Parkin-mediated mitophagy, removes damaged mitochondria, restores mitochondrial morphology and ATP production, reduces ROS overproduction and inflammation, upregulates contractile phenotype proteins, and alleviates bladder injury and urinary dysfunction in DBD rats. These beneficial effects were abolished by AMPK inhibitors, confirming that restoring mitochondrial bioenergetics effectively ameliorates DBD (Figure 3).

Mitochondrial transplantation also contributes to neural function recovery, which is essential for normal micturition reflexes. Yao et al. [58] revealed that mitochondrial transfer from mesenchymal stem cells (MSCs) reduces neuronal ferroptosis, preserves neuronal morphological integrity, increases the proportion of Nissl body-positive neurons, inhibits microglial hyperactivation and astrogliosis, reduces neuroinflammation and glial scar formation, improves the microenvironment for neural regeneration, and ultimately promotes motor function recovery after spinal cord injury. Chandrasekaran et al. [59] found that NAD^+^ precursors (NMN/NR) restored mitochondrial respiratory capacity in dorsal root ganglion neurons, improved nerve conduction velocity, alleviated hyperalgesia and mechanical allodynia, reduced intraepidermal nerve fiber loss, attenuated oxidative stress and axonal degeneration, and ameliorated diabetic peripheral neuropathy and bladder dysfunction. Although direct evidence supporting mitochondrial transplantation for bladder innervation repair in DBD remains limited, these findings strongly suggest its promising therapeutic value (Figure 3).

Mitochondrial dysfunction disrupts calcium homeostasis by impairing mitochondrial calcium uptake and release, as well as reducing ATP supply for calcium pumps and channels. Boutonnet et al. [60] demonstrated that ischemia/reperfusion (IR) injury reduced mitochondrial calcium retention capacity and induced calcium overload, leading to premature opening of the mitochondrial permeability transition pore (mPTP). Autologous mitochondrial transplantation partially restored calcium retention capacity, delayed abnormal mPTP opening, repaired respiratory function, provided sufficient energy for calcium handling, and relieved skeletal muscle injury. Li et al. [61] found that in cardiomyocytes with SLC25A3 deficiency, transplantation of healthy mitochondria enhanced respiratory chain activity, increased ATP production, reduced lactic acid and H^+^ accumulation, prevented calcium influx mediated by Na^+^/H^+^ and reverse Na^+^/Ca^2+^ exchangers, balanced calcium transport and storage, reduced cytosolic calcium overload, and improved myocardial contractile function. In DBD, hyperglycemia-induced mitochondrial damage causes calcium dyshomeostasis, leading to detrusor contractile disorder, apoptosis, and fibrosis. Mitochondrial transplantation restores mitochondrial calcium buffering capacity, delays aberrant mPTP opening, enhances the ATP supply for calcium-regulating proteins, stabilizes intracellular calcium balance, inhibits calcium-dependent injury signaling, and improves detrusor coordination, thereby alleviating urinary dysfunction (Figure 3).

Although mitochondrial transplantation shows multifaceted potential for restoring bladder function in DBD by suppressing oxidative stress, restoring energy supply, protecting innervation, and maintaining calcium homeostasis, several critical limitations and challenges remain. First, direct in vivo evidence in DBD models is scarce, as most studies are derived from other organ injury systems, lacking DBD-specific validation and quantitative efficacy data. Second, the detailed molecular mechanisms remain incompletely clarified, especially regarding how mitochondrial transplantation regulates nerve–smooth muscle crosstalk, calcium signaling, and fibrogenesis in DBD. In addition, although restoring mitochondrial function can improve bladder function in diabetic bladder disease through the aforementioned mechanisms, it remains unclear whether mitochondrial transplantation can recapitulate these therapeutic effects. Therefore, extensive experimental validation is still required to confirm this hypothesis. Future research should establish DBD-specific mitochondrial transplantation models; optimize delivery routes, doses, and timing; develop targeted mitochondrial delivery systems to improve bladder tropism and survival; evaluate the safety and immunogenicity of allogeneic and autologous mitochondria; and explore combination strategies with genetic modification or small-molecule adjuvants to enhance therapeutic efficacy. These efforts will accelerate the clinical translation of mitochondrial transplantation as a novel and effective strategy for DBD (Table 1).

## 4. Therapeutic Strategies, Comparison, Innovation, and Challenges of Mitochondrial Transplantation in DBD

### 4.1. Comparison of Mitochondrial Transplantation and Traditional Therapeutic Approaches

Traditional therapeutic strategies for DBD are generally centered on the principle of “controlling the underlying disease + improving bladder function + preventing complications”, involving four modalities: basic intervention, pharmacotherapy, physical therapy, and surgical treatment [67,68,69]. For instance, the underlying disease is managed via oral hypoglycemic agents or insulin injections [70]; medications are administered to improve detrusor function, repair neural function, and prevent infections [67,71]; electrical stimulation is employed to restore pelvic floor muscle and nerve function [72]; and for severe cases, interventions such as catheterization and urinary diversion are implemented to empty residual urine, thereby protecting renal function [73]. As an emerging therapeutic strategy, mitochondrial transplantation aims to restore normal cellular function by supplementing or replacing impaired mitochondria. It has demonstrated unique advantages in the treatment of other diseases. For example, in myocardial IR injury, traditional treatments primarily focus on restoring blood flow and administering medications to alleviate damage, whereas mitochondrial transplantation can directly replenish mitochondria in injured cardiomyocytes, enhance myocardial function, and reduce infarct size [30]. In Parkinson’s disease research, although numerous therapeutic strategies targeting mitochondrial dysfunction have been developed, most yield suboptimal clinical outcomes; mitochondrial transplantation is expected to provide a novel therapeutic avenue by improving mitochondrial function in neurons [74]. Compared with traditional therapies, mitochondrial transplantation offers greater specificity and can ameliorate disease states at the source of cellular energy metabolism. However, it still faces multiple challenges, such as optimizing mitochondrial sources and transplantation techniques. In contrast, traditional therapeutic methods possess the advantage of abundant clinical application experience. Future research should comprehensively consider the strengths and limitations of both approaches to explore more effective treatment regimens (Table 2).

### 4.2. Research on Combined Therapy Regimens of Mitochondrial Transplantation

To enhance therapeutic efficacy, research on mitochondrial transplantation combined with other therapeutic modalities has attracted increasing attention. In studies of myocardial IR injury, the combined administration of coenzyme Q10 (CoQ10) and mitochondrial transplantation exerted a synergistic effect in improving myocardial function in aged rats, reducing cardiac troponin I (cTn-I) levels, regulating autophagic activity, and decreasing the production of proinflammatory cytokines, which yielded more pronounced outcomes than monotherapy [81]. In research on premature ovarian insufficiency, the combined application of mesenchymal stem cell-derived mitochondria (MSC-Mito) and pyrroloquinoline quinone (PQQ) significantly restored ovarian function and antioxidant capacity in mice, reduced follicular loss, and ameliorated estrous cycle disorders via the SIRT1/ATM/p53 signaling pathway [82]. In the treatment of prostate cancer and ovarian cancer, mitochondrial transplantation can enhance chemosensitivity, reduce the effective drug dosage, promote tumor cell apoptosis by downregulating anti-apoptotic proteins (p-AKT, p-Bad, p-Bcl-2) and upregulating pro-apoptotic proteins (p53, p-JNK), and regulate apoptotic signaling pathways, thereby improving the anti-tumor efficacy against prostate and ovarian cancers [83]. For the treatment of DBD, these strategies can be adopted for reference to explore combined regimens of mitochondrial transplantation with antioxidants, neurotrophic factors, and other agents, so as to enhance therapeutic effects. Meanwhile, in-depth investigations into the synergistic mechanisms of combined therapy are required to provide a more solid theoretical foundation for its clinical application (Table 1).

### 4.3. Delivery Routes of Mitochondrial Transplantation in the Treatment of Diabetic Bladder Dysfunction

The delivery routes of mitochondrial transplantation for DBD are currently mainly derived from experience in cardiovascular and renal injury models. Based on the core pathological features of DBD—mitochondrial dysfunction in bladder smooth muscle and urothelium, accompanied by local inflammation and fibrosis—three therapeutic strategies have been proposed: local administration, systemic circulatory delivery, and cell-mediated delivery [84,85,86]. As the most recognized core strategy, local intravesical administration enables precise delivery of healthy mitochondria to injured tissues while avoiding systemic loss. It includes two main approaches: Transurethral intravesical infusion, a minimally invasive and repeatable technique in which mitochondrial suspension is instilled into the bladder via a catheter and retained. This approach targets the urothelium and superficial smooth muscle layers and helps overcome the challenges of poor mitochondrial penetration across the epithelial barrier and dilution by urine. Local injection into the bladder wall under ultrasound or laparoscopic guidance allows direct delivery into the smooth muscle layer, enabling rapid uptake by damaged cells and higher repair efficiency. This method is particularly suitable for patients with advanced DBD who present with bladder smooth muscle fibrosis and impaired contractility [87,88]. For DBD patients with systemic mitochondrial dysfunction and pelvic microcirculatory disturbances secondary to diabetes, internal iliac artery perfusion serves as a supplementary systemic strategy. Mitochondria are infused through the main blood supply of the bladder, reaching bladder tissue via blood flow while potentially benefiting other pelvic organs. However, this route shows relatively low targeting efficiency, as mitochondria are prone to clearance by the liver and spleen, requiring bladder-specific antibody-modified nanoparticles to improve enrichment [89]. Furthermore, cell-mediated mitochondrial delivery represents an important refined strategy. In this indirect transplantation approach, healthy mitochondria are loaded into mesenchymal stem cells ex vivo. The engineered stem cells are then delivered to lesions via intravesical instillation or local injection. Through stem cell homing, precise mitochondrial transfer to injured cells is achieved. Meanwhile, the stem cells secrete anti-inflammatory and anti-fibrotic factors, which concurrently improve the bladder microenvironment, matching the dual pathological features of mitochondrial dysfunction and inflammation-fibrosis in DBD.

Current research still faces notable limitations. The key challenges include improving the survival, engraftment, and functional integration of transplanted mitochondria within injured bladder cells. Moreover, as a chronic progressive disorder, DBD is unlikely to achieve long-term restoration with a single transplantation. Future studies will focus on nanocarrier optimization for local formulations and combination therapies targeting bladder microenvironment improvement to further enhance targeting and efficacy. Safe and convenient repeatable administration regimens will also be explored to promote the clinical translation of mitochondrial transplantation for DBD.

### 4.4. Personalized Mitochondrial Transplantation Strategies for the Treatment of DBD

Personalized mitochondrial transplantation therapy has also been widely applied in current clinical research. In cardiomyocytes derived from Barth syndrome patients (BTHS CMs), allogeneic iPSC-differentiated normal cardiomyocytes were introduced. Fluorescent labeling tracking confirmed the colocalization of donor and recipient mitochondria. The results demonstrated that this treatment significantly improved mitochondrial function in BTHS CMs. Moreover, it stimulated the maturation of BTHS CMs, upregulated the expression of myocardial markers (cTnT, cTnI, and MLC2v), ameliorated sarcomere structural disorganization, and ultimately alleviated BTHS-associated myocardial dysfunction [90]. Other studies have shown that urine cells from Leber’s hereditary optic neuropathy (LHON) patients with mtDNA mutations were reprogrammed into iPSCs, which were further differentiated into nestin-positive neural progenitor cells (NPCs). These LHON NPCs were then co-cultured with MSCs and purified via puromycin selection. Post-treatment assessments indicated marked improvements in mitochondrial metabolic function, restoration of basal respiration and ATP production capacity in subsequently differentiated neurons, and effective alleviation of LHON-related mitochondrial functional deficits and neuronal electrophysiological abnormalities [91]. Personalized mitochondrial transplantation strategies hold profound significance for the treatment of DBD. Notably, individual patients exhibit distinct variations in diabetes subtype, disease duration, complication profiles, and genetic backgrounds—factors that may modulate the therapeutic efficacy of mitochondrial transplantation. For instance, patients carrying the mtDNA A3243G mutation may present with multiple symptoms including neurogenic bladder in addition to diabetes. For such patients, mitochondrial transplantation protocols should take into account the impact of the mutation on mitochondrial function, with careful selection of appropriate donor mitochondria and optimized transplantation regimens [92]. In Wolfram syndrome patients, mutations in the *WFS1* gene cause mitochondrial dysfunction, which in turn leads to diabetes, optic atrophy, and potentially bladder dysfunction [93]. Targeted personalized mitochondrial transplantation strategies for this cohort should integrate their specific genetic background to precisely select donor mitochondria capable of correcting mitochondrial functional deficits, while optimizing transplantation techniques to enhance treatment specificity and effectiveness. Additionally, it is imperative to account for individual patient variables such as age and physical status when formulating personalized treatment plans, thereby maximizing therapeutic outcomes (Table 1).

### 4.5. Innovations, Technical Bottlenecks and Solutions in Mitochondrial Transplantation

Mitochondrial transplantation technology has been continuously improved and innovated. For example, super donor cells are constructed by transfecting MSCs with CD38 plasmids to secrete mitochondrial-rich extracellular vesicles (Super-EV-Mito). The mitochondrial yield of Super-EV-Mito is three times higher than that of Ctrl-EV-Mito derived from ordinary MSCs, and its mitochondria exhibit superior functionality, thereby enhancing the efficiency and accuracy of experiments [38]. In nerve injury models, sciatic nerve explants were co-cultured with mitochondria to determine the optimal dosage for local mitochondrial injection. Subsequent local injection in a rat model of sciatic nerve crush injury demonstrated that mitochondrial transplantation improved the animals’ neurological behaviors, nerve conduction electrophysiology, and muscle activity; reduced oxidative stress; and increased the expression of neurotrophic factors [94]. In cellular models, normal mitochondria isolated from human umbilical cord MSCs, human umbilical vein endothelial cells, and human embryonic kidney cells were transferred into prostate cancer cells (PC-3). The results showed that this intervention promoted cell proliferation and rescued cisplatin-induced cell death. Notably, it did not affect erastin-induced ferroptosis; instead, it enhanced erastin-mediated ferroptosis in PC-3 cells, providing novel insights for cancer therapy [95]. Furthermore, the technology of single-cell mitochondrial transplantation using a nanosyringe achieves a success rate of 95%, and the transferred mitochondria can integrate into the host mitochondrial network, offering a new tool for mitochondrial research and therapy [96]. These technological innovations have expanded the potential applications of mitochondrial transplantation in the treatment of DBD.

However, mitochondrial transplantation still faces several technical bottlenecks. First and foremost, improving mitochondrial purity is a critical priority. Currently, modifications to buffers and the addition of filtration steps based on the traditional combination of differential centrifugation, density gradient centrifugation, and filtration methods can enhance mitochondrial purity. Nevertheless, these optimizations concomitantly increase time, labor, and financial costs, meaning that the refinement of purification protocols remains a key challenge to be addressed in future research. Second, the mechanisms underlying intercellular mitochondrial transfer are not fully elucidated. Although several potential pathways have been identified, such as tunneling nanotubes, extracellular vesicles, and gap junction channels, the specific regulatory mechanisms await further in-depth investigation [97]. Additionally, achieving efficient and precise delivery of mitochondria to target cells represents a major hurdle. Traditional co-culture methods are inefficient, whereas physical approaches like MitoPunch can improve efficiency but involve relatively complex operations [98]. To address these issues, researchers have been actively exploring novel strategies. For instance, a magnetomitotransfer method has been developed: mitochondria are labeled with anti-TOM22 magnetic beads and guided by magnetic plates, enabling more efficient and rapid mitochondrial transfer into recipient cells, with the transferred mitochondria retaining functional activity [99]. In addition, mitochondrial modification (e.g., O-GlcNAcylation) can enhance the stability and functionality of mitochondria in the extracellular environment, thereby boosting transplantation efficacy [32]. Researchers have also utilized photobiomodulation (PBM, i.e., near-infrared laser therapy) to upregulate the expression of the neuron-specific connexin Cx36, facilitating the transfer of exogenous mitochondria into neurons, improving the targeted efficacy of mitochondrial transplantation, and promoting neural repair [100]. Further technological optimizations are required in the future to improve the efficiency and precision of mitochondrial transplantation (Table 1).

## 5. Discussion

Mitochondrial transplantation is liable to give rise to multiple controversies in its clinical application. In terms of technology, as an emerging industrial technology, it suffers from a lack of standardized evaluation systems for its preparation, operation and efficacy assessment, which results in poor consistency of research outcomes. There is no unified standard for its dose–effect relationship, leading to blind dose selection; additionally, the relationship exhibits non-linear characteristics and significant individual differences due to the influence of disease stages and host physiological status. Mitochondria have a wide biodistribution in the body, which leads to poor targeting ability. Moreover, their inability to easily penetrate biological membranes and high susceptibility to recognition by the immune system render them prone to clearance by non-target tissues. Furthermore, the molecular mechanism underlying the functional integration of transplanted mitochondria with the host mitochondrial network remains incompletely elucidated, which remains a critical issue requiring in-depth investigation. Although the short-term acute safety of mitochondrial transplantation has been verified in animal experiments, large-sample research and long-term monitoring data for its long-term safety are lacking, leaving the long-term impacts of mitochondrial transplantation on the stability of the host genome and mitochondrial network unclear. The major potential safety hazard is the risk of inflammatory responses: for instance, damage-associated molecular pattern (DAMP) molecules inherent to mitochondria and the immunogenicity of allogeneic mitochondria can be recognized by the host immune system, thereby activating inflammatory signaling pathways. In chronic inflammatory diseases such as DBD, the local microenvironment can amplify such inflammatory responses and exacerbate tissue damage. In terms of commercial manufacturing, the fuzzy regulatory classification of mitochondrial transplantation has resulted in the absence of exclusive approval criteria for this technology. It is currently tentatively classified as a cell therapy product or biological product for regulatory purposes, which leads to a complicated approval process for its clinical trials. Meanwhile, the inherent characteristics of mitochondria as living organelles pose great challenges to the large-scale and automated production of mitochondria in manufacturing. Coupled with the immature in vitro storage and transportation technologies, the production costs of mitochondrial transplantation remain exorbitantly high. In clinical application, there is a lack of personalized treatment regimens tailored to disease characteristics and individual patient conditions. Local delivery operations demand high-level interdisciplinary professional capabilities, and both clinicians and patients hold a low acceptance of this technology due to the lack of large-sample evidence validating its long-term efficacy and safety. Looking ahead, mitochondrial transplantation technology is expected to advance in multiple aspects. First and foremost, a full-chain standardized system should be established. For example, standardization should be implemented across four key links, namely donor selection, preparation operation, transplantation implementation and efficacy assessment, to address the issue of poor consistency in experimental results. Simultaneously, universal quality control and quality assurance standards for the field should be formulated. Only by unifying industry standards first can we further resolve the ambiguity in the regulatory classification of mitochondrial transplantation technologies and enhance the consistency and recognition of their clinical therapeutic effects. In addition, the targeting ability of mitochondrial transplantation technology needs to be strengthened. Given the individual and disease-specific differences, as well as the wide biodistribution of mitochondria in the body, improving the targeting efficiency and tissue penetration of transplanted mitochondria to target tissues is the key to advancing this technology. This can be achieved through tissue-specific molecular modification of the mitochondrial surface, precise optimization of delivery routes and modification of the local microenvironment. Furthermore, O-GlcNAcylation modification of the mitochondrial surface can reduce the recognition and clearance by the mononuclear phagocyte system in the liver and spleen, thereby increasing the concentration of mitochondria in target tissues following systemic delivery. To prevent potential inflammatory responses induced by mitochondrial transplantation, the priority should be given to the use of autologous mitochondria or mitochondria from closely related donors to minimize the risk of such reactions. Nevertheless, ethical principles such as non-maleficence and beneficence must be strictly abided by during the acquisition of such mitochondria. In addition, membrane coating technology can be adopted to wrap mitochondria with autologous red blood cell membranes or mesenchymal stem cell-derived exosome membranes, which shields DAMP molecules such as mtDNA and phosphatidylserine, avoids recognition by host pattern recognition receptors (PRRs) and thus reduces inflammatory responses caused by damaged mitochondria. In terms of production, the establishment of the aforementioned standardized system must be completed first. On this basis, subsequent advancements can be realized, including the large-scale and serum-free culture of donor cells, the automated and continuous production of mitochondrial isolation and purification, the development of real-time quality inspection systems, the optimization of long-term storage conditions and safe transportation solutions, as well as subsequent improvements to reduce costs and enhance efficiency. It is believed that with the in-depth study of mitochondrial biology, more disease subtypes suitable for mitochondrial transplantation treatment will be identified in the future, expanding its application scope. At the same time, interdisciplinary collaboration should be strengthened, and the knowledge of biology, medicine, engineering and other disciplines should be comprehensively applied to drive the innovative development of mitochondrial transplantation technology. Future research hotspots for DBD treatment will focus on the in-depth exploration of its pathogenesis. Further clarifying the interactions among various pathological processes such as oxidative stress, inflammatory responses, and apoptosis, as well as their associations with mitochondrial dysfunction, will help identify novel therapeutic targets. For example, investigating the mechanism of action of the NLRP3 inflammasome in DBD may provide new directions for treatment [26]. Meanwhile, developing more effective therapeutic methods is also a research priority. Beyond optimizing mitochondrial transplantation technology, combined treatment regimens will be explored, such as the integration of mitochondrial transplantation with pharmacotherapy and gene therapy, to enhance therapeutic outcomes. In addition, leveraging artificial intelligence and big data technologies for accurate subtyping and personalized treatment of DBD patients—formulating targeted therapeutic strategies based on patients’ genetic information and clinical characteristics—represents a future development trend. Besides treatment, prevention is also an important approach to reduce the incidence of DBD. For the diabetic population, whether DBD and even a series of susceptible complications can be prevented in advance through early mitochondrial screening and transplantation will become a major direction for the application of mitochondrial function in the medical care of DBD patients. Although this approach currently faces problems such as invasiveness and insufficient specificity and sensitivity of indicators, technical optimization can be achieved by further developing oxidative stress-related indicators in urine or blood, mtDNA fragments, and high-sensitivity detection technologies. Through these research efforts, more effective therapeutic methods can be developed for DBD patients to improve their quality of life.

## Figures and Tables

**Table 1:** Mechanistic pathways and therapeutic outcomes of mitochondrial transplantation across disease models.

Mechanism of Action	Core Pathways/Targets	Major Diseases/Models	Key Therapeutic Effects	References
Antioxidant Stress Inhibition	ROS, NO, SOD, GSH, Nrf2/HO-1, MDA	Alzheimer’s disease, cerebellar degeneration, tendon injury, AKI	Reduces ROS levels, enhances antioxidant capacity, and alleviates inflammation, apoptosis, and fibrosis.	[21,55,62,63]
Restoration of Mitochondrial Energy Metabolism	OXPHOS, ATP, respiratory chain complexes, COX	Retinal injury, inflammatory myopathy, depression, schizophrenia	Restores energy supply, improves smooth muscle contraction/viability, and enhances neurological function.	[56,57,64]
Restoration of Neural Function	Ferroptosis, glial cell activation, neuroregeneration, neural signaling	Spinal cord injury	Protects neurons, suppresses neuroinflammation, and promotes nerve repair.	[58,65]
Correction of Calcium Homeostasis Imbalance	mPTP, calcium retention, calcium pumps/channels, Ca^2+^ transients	Skeletal muscle IR injury, cardiac hypertrophy	Reduces calcium overload, restores smooth muscle contractility, and inhibits mitochondria-dependent tissue damage.	[61,66]

**Table 2:** Comparison of core characteristics of different therapeutic approaches for DBD.

Therapy Type	Core Logic	Representative Methods	Advantages	Limitations	References
Conventional Therapy	Control of underlying diseases and symptomatic intervention	Hypoglycemic drugs/insulin, detrusor modulators, electrical stimulation, catheterization	Rich clinical experience, non-invasive/minimally invasive	Weak targeting; failure to reverse mitochondrial damage	[71,75,76]
Single Mitochondrial Transplantation	Restoring energy metabolism at the organelle level	Targeted delivery of mesenchymal stem cell-derived mitochondria	Directly targeting pathological core, multi-target repair	Difficulties in purity control; room for improvement in targeted delivery efficiency	[77,78]
Combination Therapy	Synergistic enhancement and precise matching	Mitochondrial transplantation combined with antioxidants, neurotrophic factors, or chemotherapy	Enhanced therapeutic efficacy; reduced dosage of single therapy	Requiring individualized regimen design	[79,80]

## Data Availability

No new data were created or analyzed in this study. Data sharing is not applicable to this article.
